# Spontaneous Resolution of Primary Hyperparathyroidism in Parathyroid Adenoma

**DOI:** 10.1155/2012/793753

**Published:** 2012-10-30

**Authors:** Sara J. Micale, Michael P. Kane, Robert S. Busch

**Affiliations:** ^1^Department of Pharmacy Practice, Albany College of Pharmacy and Health Sciences, 106 New Scotland Avenue, Albany, NY 12208, USA; ^2^The Endocrine Group, LLP, Albany, NY 12206, USA

## Abstract

A 71 yo woman with primary hyperparathyroidism awaiting surgery because of significant hypercalcemia and hypercalciuria presented to the local emergency department with the chief complaints of discomfort in her neck, sore throat, and difficulty swallowing. She was found to be hypocalcemic with a calcium level of 8.1 mg/dL. She was seen by her endocrinologist three days later at which time serum calcium, iPTH, and serum phosphate levels were all within normal limits. Based on history and a series of ultrasounds the patient was diagnosed with spontaneous infarction of her parathyroid adenoma, which resulted in resolution of her primary hyperparathyroidism.

## 1. Introduction

Primary hyperparathyroidism typically presents as hypercalcemia in an otherwise asymptomatic patient and is most frequently caused by a single parathyroid gland adenoma (approximately 85% of cases) [1]. The American Association of Clinical Endocrinologists recommends parathyroidectomy in asymptomatic patients who are less than fifty years of age, in patients unavailable for appropriate followup, in those with a serum calcium level >1.0 mg/dL above normal, in patients with a urinary calcium level >400 mg/24 h, in patients with a 30% or more decrease in renal function, and in patients with complications of primary hyperparathyroidism such as nephrocalcinosis, osteoporosis, or a severe psychoneurologic disorder [1]. Medical management with bisphosphonates, estrogen, or cinacalcet is options in nonsurgical candidates [1]. In rare instances, patients have autoinfarcted the affected gland with subsequent spontaneous resolution of the disease. We report such a case.

## 2. Case Report

On July 21, 2012 a 71-year-old Caucasian woman with primary hyperparathyroidism awaiting surgery because of significant hypercalcemia and hypercalciuria presented to her local emergency department after acute onset of discomfort within her neck that ran transversely from left to right from the mid to lower neck, sore throat, and difficulty swallowing that had progressively gotten worse over the previous 3 days. She denied chest pain, jaw pain, arm pain, fever, or vomiting. Her past medical history included primary hyperparathyroidism, diagnosed eight months previously, which was due to a left sided parathyroid adenoma. The patient was subsequently scheduled for parathyroid surgery. Prior to this acute event, her parathyroid hormone had been elevated, with concurrent hypercalcemia, hypophosphatemia, and an increased ionized calcium level (Table 1). 

A CT scan performed in the emergency department showed a 2.1 × 2.4 × 3.6 cm soft tissue mass posterior to the left thyroid, lateral to the esophagus, and abutting the anterior aspect of the C5-C6 vertebral bodies. Localization of this adenoma was consistent with previous findings resulting from a sestamibi scan. Laboratory data in the emergency department revealed hypocalcemia (8.1 mg/dL), a negative rapid strep test, and a normal white blood cell count (6.6/mm^3^). The patient was discharged with a diagnosis of retropharyngeal mass and instructed to followup with a surgeon in two days.

Three days following emergency department discharge (July 24, 2012), the patient reported to her endocrinologist for followup. An ultrasound performed that day reported a soft tissue mass posterior to the left lobe of the thyroid measuring 3.3 × 1.2 × 1.3 cm, results consistent with a parathyroid adenoma. Follow-up laboratory data revealed normal calcium, PTH, and phosphate levels of 8.77 mg/dL, 64.3 pg/mL, and 2.71 mg/dL, respectively. A second ultrasound performed 16 days after the emergency department visit demonstrated a decrease in the mass to 2.0 × 0.9 × 0.7 cm. Based on the patient's reported symptoms, sudden decrease in PTH and calcium levels, and decrease in size of the adenoma, the patient was diagnosed with infarction of her left lower parathyroid adenoma. The scheduled parathyroid surgery was canceled, and, because of the possibility of parathyroid adenoma recurrence, the patient was counseled to continue routine followup with her endocrinologist. 

## 3. Discussion

Spontaneous infarction of a parathyroid adenoma is an uncommon event that may result in the resolution of primary hyperparathyroidism [2]. Norris first documented autoinfarction of a parathyroid gland in a case report in 1946 [3]. 

The patient presenting in the current case had a diagnosis of primary hyperparathyroidism for approximately eight months previous to the adenoma infarction. Her symptoms were nonspecific upon arrival to the emergency department and it was not until laboratory data and multiple ultrasounds were interpreted that a diagnosis of parathyroid adenoma infarction was made. The comparative changes in the size of the adenoma are listed in Table 2. 

The patient's dysphagia, neck discomfort, and sore throat are typical symptoms of a parathyroid gland infarction [2]. The literature reports pain, swelling, and tenderness in the anterior neck, dysphagia, hoarseness, and ecchymosis as common occurrences upon presentation of an infarcted cervical parathyroid gland [2, 4] though some patients are reportedly asymptomatic when experiencing such an event [4]. 

**Figure 1:**
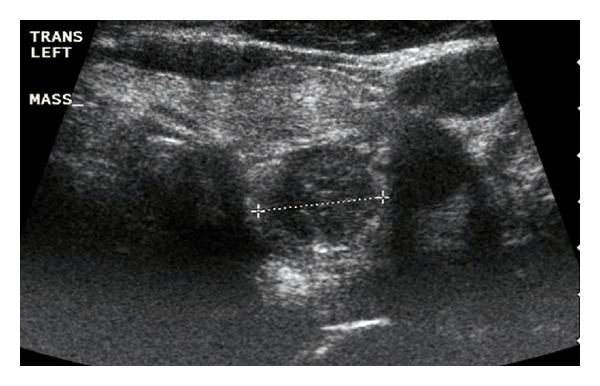
Ultrasound number 1 of left lower parathyroid gland—7/24/12: after infarction.

**Figure 2:**
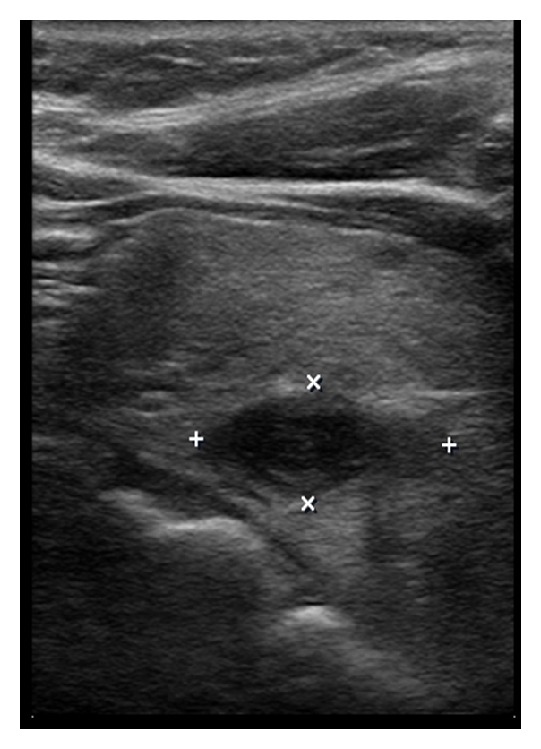
Ultrasound number 2 of left lower parathyroid gland—8/9/12: after infarction.

The CT scan at the emergency department documented the exact size of the adenoma at the time of the presenting symptoms. The ultrasound three days following the emergency room visit documented that the adenoma increased in size (Figure 1). This has been previously reported in the literature, with the presumption that the increase in size is the result of a hemorrhagic infarction [2]. The subsequent decrease in adenoma size status after the emergency department visit, as shown in the second ultrasound (Figure 2), is consistent with infarction. One case report of parathyroid adenoma infarction reported by Kovacs and Gay in 1998 actually demonstrated complete disappearance of the parathyroid adenoma [5]. 

Short-term remission of hypercalcemia is often seen after an acute infarction of a parathyroid adenoma [2], though patients may have a recurrence of primary hyperparathyroidism at a later date [5]. Only 12 cases of long-term complete remission have been documented [5]. It is recommended that clinical and laboratory followup be continued for at least one year after infarction in order to monitor for the recurrence of primary hyperparathyroidism [6] though, recurrence as late as three years out has been reported [7]. Although no specific guideline recommendation exists regarding the duration and frequency of followup, it is reasonable to continue monitoring these patients for clinical, biochemical, and imaging changes indefinitely. We plan to follow this patient every six months to monitor for biochemical and/or symptomatic recurrence of disease.

## 4. Conclusion

Spontaneous infarction of a parathyroid gland is a rare occurrence. The rate of recurrence of primary hyperparathyroidism after an adenoma infarction is unknown though its recurrence is well documented. Because of the possibility of symptomatic recurrence following infarction of a parathyroid adenoma, it is imperative that patients who have infarcted a parathyroid adenoma continue to be appropriately monitored. 

## Figures and Tables

**Table 1:** Laboratory findings indicating hyperparathyroidism.

Lab test	Normal value	After infarction		Prior to infarction			
		9/27/12	7/24/12	6/25/12	3/14/12	1/17/12	11/25/11
Serum calcium	8.6–10.2 mg/dL	8.83	8.77	11.57	10.93	11.82	11.23
Serum phosphate	2.5–4.5 mg/L	3.21	2.71	3.08	2.45	3.58	2.82
iPTH	12–65 ng/dL	68	64.3			108	144
Ionized calcium	4.5–5.6 mg/dL					6.7	6.4

**Table 2:** Size of the adenoma.

	After infarction		Prior to infarction
	8/9/12 (US)	7/24/12 (US)	1/17/12 (CT)
cm × cm × cm	2.0 × 0.9 × 0.7	3.3 × 1.2 × 1.3	2.1 × 2.4 × 3.6
